# Supplementary material for: What does patient feedback reveal about the NHS? A mixed methods study of comments posted to the NHS Choices online service
Source: BMJ Open. 2017 Apr 27;7(4):e013821. doi: 10.1136/bmjopen-2016-013821 (PMC5566587; doi:10.1136/bmjopen-2016-013821)
Supplement: Author's manuscript [file bmjopen-2016-013821.draft_revisions.pdf]

BMJ Open

What does patient feedback reveal about the NHS? A mixed methods study of comments posted to the NHS Choices Online service

Journal:	*BMJ Open*
Manuscript ID	bmjopen-2016-013821
Article Type:	Research
Date Submitted by the Author:	09-Aug-2016
Complete List of Authors:	Brookes, Gavin; Lancaster University, Linguistics and English Language<br>Baker, Paul; Lancaster University, Linguistics and English Language
**Primary Subject Heading**:	Communication
Secondary Subject Heading:	Health services research
Keywords:	Patient feedback, NHS Choices, Quantitative linguistics

SCHOLARONE™
Manuscripts

For peer review only

Title page

Title

What does patient feedback reveal about the NHS? A mixed methods study of comments posted to the NHS Choices Online service

Authors' names

Gavin Brookes, Paul Baker

Address for each author

Gavin Brookes
Senior Research Associate
ESRC Centre for Corpus Approaches to Social Science (CASS), Department of Linguistics and English Language, Lancaster University, Lancaster, UK, LA1 4YL

Paul Baker
Professor of Linguistics and English Language
ESRC Centre for Corpus Approaches to Social Science (CASS), Department of Linguistics and English Language, Lancaster University, Lancaster, UK, LA1 4YL

Corresponding author

Gavin Brookes
ESRC Centre for Corpus Approaches to Social Science (CASS)
Department of Linguistics and English Language
Faculty of Arts and Social Sciences Building
Lancaster University
Lancaster
UK
LA1 4YL
g.brookes@lancaster.ac.uk

For peer review only

Abstract

Objective

To examine the key areas of concern expressed by patients giving feedback on NHS services in England and to understand the specific issues within these areas of concerns and how they drive positive and negative evaluation.

Design

Computer-assisted quantitative and qualitative study of 228,113 comments (28,971,142 words) of feedback posted to the NHS Choices website.

Participants

Contributors posting comments about the NHS between March 2013 and September 2015.

Results

Overall, NHS services were evaluated positively approximately three times more often than negatively. Four key areas of focus were: Treatment, System/Organisation, Communication and Interpersonal Skills. Treatment exhibited the highest proportion of positive evaluative comments (88%), followed by System/Organisation (51%), Communication (40%) and then Interpersonal Skills (18%). Qualitative analysis revealed that reference to staff interpersonal skills featured prominently, even in comments relating to Treatment and System/Organisational issues. Positive feedback was elicited in cases of staff being caring, compassionate and knowing patient's names while rudeness, apathy and not listening were frequent drivers of negative feedback.

Conclusions

Although technical competence is undoubtedly an important aspect of healthcare provision, staff members were much more likely to be evaluated both positively and negatively according to their interpersonal skills. Therefore, the findings reported in this study highlight the salience of such 'soft' skills to patients and emphasise the need for these to be focused upon and developed in staff training programs. The findings also reveal a significant overlap between the four key areas of concern in the ways that care is evaluated by patients, raising questions about the reliability of patient feedback exercises for evaluating the technical competence of practitioners.

Strengths and limitations of this study

- This study examines the largest (228,113 comments and approximately 29 million words) and most recent (March 2013 to September 2015) collection of patient feedback data on NHS services analysed to-date.
- Building on previous research, the feedback data examined relates to a wider range of areas of health care service provision, including: acute trusts, care organisations,

For peer review only

care providers, clinical commissioning groups, clinics, dentists, GP practices, hospitals, mental health trusts, opticians and pharmacies.

- The use of quantitative computer-assisted linguistic techniques produces large-scale, generalisable insights into this vast dataset, while more fine-grained, qualitative analysis helps to elucidate more specific patient concerns and areas of difference and overlap that have been overlooked by research employing solely quantitative approaches.
- Further data and research required to assess possible demographic trends in the feedback given.

For peer review only

Introduction

Since the 1980s, patient feedback exercises have been undertaken by an increasing number of healthcare providers worldwide in order to monitor the quality of the services they provide and stimulate improvements where needed.[1] Although the reliability of patient feedback as an indicator of the technical quality of care remains a topic of debate,[2] patient feedback exercises have nonetheless become a staple way of measuring and regulating healthcare standards and ensuring public involvement in the design and improvement of healthcare provision. In England, since 2002 patient feedback has played an increasingly significant role in the way that care quality is assessed, with all National Health Service (NHS) trusts required to collect and report the results of feedback on their services to the regulatory body, the Healthcare Commission. The importance of the insights gained from patient feedback exercises is all the more pronounced in this context, where reductions in government expenditure in areas of social provision has required healthcare providers to constantly demonstrate both the quality and financial viability of the services they provide.

Given the increasing significance of patient feedback to the ways that healthcare services are designed, delivered and regulated, there is a pressing need for research that accounts for the concerns expressed by patients in their feedback. However, rather than explore the content of patient feedback itself, the majority of existing research in this area is concerned chiefly with: reviewing the suitability of instruments and methods of collecting and analysing feedback,[3] considering the reliability of feedback data for assessing healthcare quality,[4] reflecting on the extent to which insights gained from such exercises have actually improved service provision,[5] and recommending how such insights might be translated into positive clinical outcomes in the future.[6] The comparatively few studies that have examined the content of patient feedback (even fewer of which relate to healthcare in England), have reported recurring areas of concern to include the technical quality of the care, accessibility to care and the interpersonal and communication skills of practitioners (with the latter two often conflated).[7-9]

The present study identifies and examines the key areas of concern for patients providing positive and negative feedback on healthcare services in England between March 2013 and September 2015. Our findings build on existing patient feedback research in several important ways. At 228,113 comments and approximately 29 million words, the feedback data we analyse is considerably larger than that examined in previous research on this topic, which has mainly accounted for hundreds of comments[10-12] and at the most in the tens of thousands.[13] Moreover, the data we analyse represents feedback relating to a wider range of healthcare services than considered in previous research, which has often focussed on specific areas of healthcare provision.[14] The lion's share of research on patient feedback was conducted using data collected in the 1990s and early 2000s, while our dataset contains comments made as recently as September 2015, making this dataset the most up-to-date of

For peer review only

its kind. More broadly, given the ever changing landscape of healthcare provision in England and the U.K., the present study responds to the need for regular and up-to-date research that assesses patient attitudes towards the NHS and specifically identifies the key drivers of feedback about the particular services they access.

Methods

Data

We studied the written feedback posted to the NHS Choices online service (http://www.nhs.uk/pages/home.aspx) between March 2013 and September 2015 (data made available to the researchers). The data comprises a total of 228,113 comments, amounting to 28,971,142 words. The comments relate to a variety of areas of healthcare service provision, including acute trusts, care organisations, care providers, clinical commissioning groups, clinics, dentists, GP practices, hospitals, mental health trusts, opticians and pharmacies.

Table 1. Service-by-service numerical breakdown of the comment database

Section	No of comments	Word counts	Mean words per comment
Acute trusts	1,022	159,385	156
Care organisations	6	1,164	194
Care providers	4,493	422,133	94
CCGs	1	253	253
Clinics	2,887	400,813	139
Dentists	41,958	4,306,698	103
GP practices	111,318	14,093,437	127
Hospitals	55,145	8,605,580	157
Mental health trusts	565	111,557	197
Opticians	1,734	179,493	104
Pharmacies	8,984	690,629	77
**All sections**	**228,113**	**28,971,142**	**146**

Analysis

We examined the feedback using computer-assisted methods of linguistic analysis afforded by CQPWeb,[15] an online tool that offers a range of techniques for quantitative and qualitative linguistic analysis of large collections of language data. We first identified the ten most frequently occurring linguistic markers of positive and negative evaluation across the comments. We then examined the words occurring frequently alongside these evaluative

For peer review only

items in order to identify the key areas of concern for patients when evaluating the care they receive. Following this, samples of messages (n= 100) in which each concern was evaluated positively and negatively were then closely read by the researchers to ascertain how and why these most frequent concerns featured in the positive and negative feedback.

Results

Quantitative findings

Table 2 displays the ten most frequent positive and negative evaluative words used in the comments. The comparatively higher frequencies of the positive words (total: 223,439) compared to the negative words (total: 73,363) provides a quantitative indication that the patients are more likely to evaluative the services they access positively than negatively. The positive evaluation words occur, on average, across almost three times as many comments as the negative words.

Table 2. Top ten positive and negative evaluative words in comments ranked in order of frequency

Positive			Negative		
**Word**	**Frequency**	**# of comments**	**Word**	**Frequency**	**# of comments**
good	59,237	46,192	bad	16,945	14,798
excellent	49,090	38,128	poor	15,274	12,548
great	34,298	27,772	worst	7,627	6,627
best	25,556	21,641	worse	7,289	6,447
fantastic	15,186	12,915	terrible	6,799	5,920
brilliant	11,546	10,136	awful	6,106	5,291
wonderful	10,371	8,846	appalling	4,410	4,007
amazing	9,749	8,081	disgusting	3,246	2,913
outstanding	5,019	4,277	ridiculous	3,206	3,023
exceptional	3,387	3,060	useless	2,461	2,217
**Total**	**223,439**	**18,105 (mean)**	**Total**	**73,363**	**6,379 (mean)**

CQPWeb was then used to generate lists of those words occurring frequently within the three words preceding and following the positive and negative evaluation words in table 2. The 100 most frequent words occurring alongside the positive and negative words were then thematically coded to reflect the most frequently evaluated areas of concern for patients giving feedback. Four areas emerged as frequent across the comments

For peer review only

(corresponding words in brackets): (i) Treatment (*care, treatment*, *dental*), (ii) System/Organisation (*system*, *appointment*, *time*), (iii) Communication (*communication*) and (iv) Interpersonal Skills (*attitude*). Based on the corresponding words (in brackets), we then examined how often each concern featured alongside the positive vs. negative evaluative words (figure 1).

Figure 1. Collocation of frequent concerns with positive and negative evaluation words

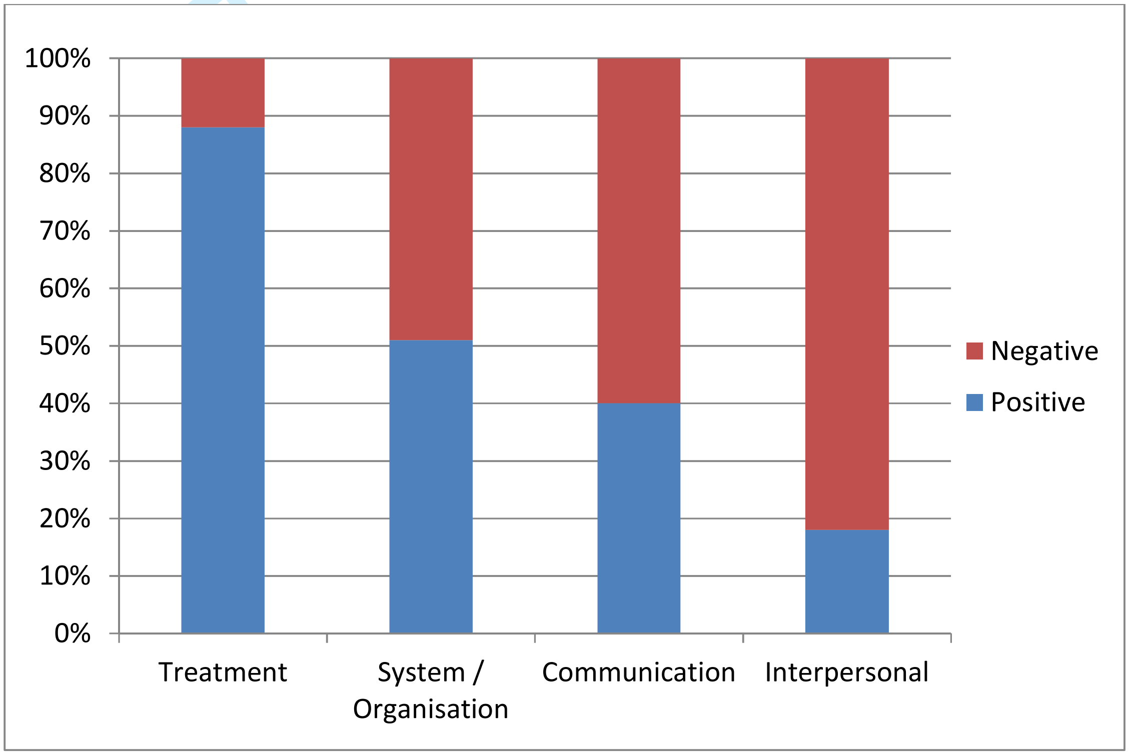

Of the four key areas of concern displayed above, Treatment exhibited the highest proportion of positive feedback, occurring alongside the positive evaluation words 88 per cent of the time. System and Organisational issues were evaluated positively (51 per cent) and negatively (49 per cent) in almost equal measure. Communication fared slightly worse in terms of evaluation, occurring alongside the negative evaluation words in 60 per cent of cases. However, Interpersonal aspects of care occurred alongside the positive words least frequently of all, just 18 per cent of the time, and so occurred alongside the negative words on 82 per cent of occasions. This table therefore suggests that while treatment fares best in terms of patient evaluation, interpersonal aspects of care fare worst.

Qualitative findings

To understand how and why the four key areas of concern were evaluated positively and negatively, we examined a sample of comments (n= 100) in which each area of concern occurred alongside the positive and then negative evaluation words.

Treatment

Treatment was most often evaluated positively in relation to the interpersonal skills of staff members (42 per cent) who were praised for being caring, compassionate, friendly, and for knowing patients' names. Treatment was evaluated in terms of technical competence (as it might be conceptualised by practitioners) in only 12 per cent of cases. In fact, treatment was just as likely to be evaluated positively on the basis of accessibility and availability of practitioners as it was those practitioners' technical competence. Almost as frequently, in 10 per cent of cases, treatment was evaluated positively if the practitioner provided care that was patient-centred, and which made patients feel involved in decisions relating to their care. Treatment was also evaluated positively if staff were perceived to be efficient (7 per cent), hard-working (6 per cent) and gave patients their time (6 per cent). In the remaining comments treatment was evaluated positively on the basis of the cleanliness of the facilities (5 per cent) and the food served during the patient's stay (3 per cent).

As with the positive comments, the quality of treatment was negatively evaluated most frequently according to interpersonal aspects of care (33 per cent), with staff members described as being apathetic and condescending, as well as lacking empathy and bullying and making fun of patients. Another frequent source of criticism in the comments relates to the lack of practitioners' availability and accessibility (25 per cent), with difficulties attaining appointments, long waiting lists, and not being able to see the same practitioner all cited as causes for complaint. Problems with technical aspects of care, including ineffective treatment and inaccurate or slow diagnoses, accounted for 18 per cent of the negative comments relating to this area of concern. Other causes for negative evaluation of treatment included breakdowns in communication between members of staff and between practitioners and patients and their families (8 per cent), a lack of aftercare following treatment (6 per cent), the uncleanliness of facilities (4 per cent), the inability to access test results (2 per cent) and a lack of seating and space in waiting areas (2 per cent).

System and organisation

The majority of positive comments concerning system and organisational issues relate in one way or another to the process of attaining an appointment with a practitioner. Of the comments which positively evaluate system issues, 25 per cent praised the booking system

used to secure an appointment. 22 per cent cited wide and flexible availability of practitioners as a positive, while 18 per cent commented positively on being able to secure an emergency appointment at short notice. Other reasons for positive evaluation in the comments included short on-site waiting times (12 per cent), as well as effective time management in relation to keeping appointments (8 per cent), efficient prescription systems (8 per cent) and useful telemedicine services (7 per cent).

Similarly, when system and organisation is evaluated negatively, it is also most frequently due to difficulties experienced in relation to appointment booking systems (27 per cent), appointment waiting times (17 per cent) and on-site waiting times (13 per cent). Other common causes for complaint include telephone waiting times (11 per cent), the lack of availability of out of hours appointments (9 per cent), poor time management in relation to keeping appointments (4 per cent) and the lack of availability of emergency appointments at short notice (4 per cent).

Communication

Just as treatment was most frequently evaluated both positively and negatively according to staff members' interpersonal skills, so too was communication. In 34 per cent of the comments in which communication was evaluated positively, patients complemented staff for their politeness, 'people skills', sense of humour and for smiling and knowing patients' names. Communication was also rated positively when it was perceived to be efficient between staff members or across departments (11 per cent), as well as when staff communication with patients was clear (9 per cent), detailed (8 per cent), frequent (i.e. with patients kept up-to-date) (7 per cent), not rushed (7 per cent), facilitated patients' perspectives (7 per cent), involved patients' families (6 per cent), seen as honest (5 per cent), sensitive (4 per cent) and accommodating to patients who are hard of hearing (1 per cent).

However, throughout the comments, communication tended to be evaluated negatively (60 per cent) more often than positively. Frequent causes for negative evaluation of communication included poor inter-staff and inter-departmental communication (particularly at staff change or handover) (23 per cent), lack of staff interpersonal skills (including being perceived as rude, arrogant and dismissive) (17 per cent), failure to inform patients about waits, delays and cancellations (15 per cent), failure to contact patients to arrange appointments (12 per cent) and failure to keep patients and their families updated regarding details of their health and treatment (11 per cent). Other causes for negative evaluation of communication include: failure to involve patients' relatives in communication (6 per cent), lack of communication in aftercare following treatment (5 per cent), consultations feeling rushed (5 per cent), telephone services being unreliable (4 per cent),

For peer review only

failure to inform patients of changes to their practitioner (1 per cent) and a perceived lack of adequate English language skills (1 per cent).

Interpersonal skills

The final area of concern we explore here is staff members' interpersonal skills. Staff members, including medical and non-medical staff, were evaluated positively in terms of their interpersonal skills most commonly if they exhibited qualities such as kindness, friendliness, cheerfulness and politeness (19 per cent). Members of staff were also evaluated as having a positive attitude if they were seen as hard working and efficient, particularly in high pressure situations (12 per cent). Other frequently cited qualities in the positive evaluation of interpersonal skills include being caring (11 per cent), sympathetic and compassionate (11 per cent) and, although it might not intuitively seem to be an interpersonal skill, knowledgeable (9 per cent). Other important factors motivating the positive evaluation of interpersonal skills include: a perceived willingness to help (6 per cent), being patient-centred in communication and decision-making (6 per cent), reassuring patients and putting them at ease (6 per cent), smiling (6 per cent), taking time out and not rushing patients (6 per cent), having a good sense of humour (4 per cent) and being a good listener (4 per cent).

Given that interpersonal skills, specifically the word *attitude*, tended to occur more frequently alongside the negative rather than positive evaluation words, it is perhaps more important to consider what motivated negative feedback in relation to this area of concern. The most frequently cited motivation for negative feedback relating to interpersonal skills was the impoliteness and perceived rudeness of staff members (41 per cent). Other common complaints included staff members being lazy (11 per cent), appearing apathetic towards patients' concerns (9 percent), not listening to patients (7 per cent), being abrupt (7 per cent), not answering the phone (6 per cent), appearing impositioned by patients (5 per cent), not smiling (5 per cent), failing to contact patients following treatment (4 per cent), laughing and joking at patients' expense (2 per cent) and lacking adequate English language skills (1 per cent).

Discussion

Statement of principal findings

The patient feedback analysed in this study paints a generally positive picture of healthcare services provided by the NHS in England. Our quantitative analysis of the patient comments revealed the most commonly used linguistic markers of positive evaluation occurred

For peer review only

approximately three times as often as markers of negative evaluation and, on average, across approximately three times as many comments. Concerns relating to Treatment, System/Organisation, Communication and Interpersonal Skills were identified as key to the ways that healthcare services were both positively and negatively evaluated. Of these key concerns, Treatment was evaluated positively (60 per cent) more often than negatively (40 per cent). System and Organisational issues were evaluated positively (51 per cent) and negatively (49 per cent) in roughly equal measure. The Communication Skills of staff were evaluated negatively (60) more than positively (40), and staff members' Interpersonal Skills were evaluated negatively (82 per cent) considerably more often than positively (18 per cent). Our analysis therefore suggests that while treatment-related issues fare well in patient feedback, staff members' interpersonal and communication skills fare less well and should be targeted as areas for improvement for NHS healthcare provision in England.

Qualitative examination of the data was able to reveal the more precise nature of the concerns expressed in the comments, as well as uncover overlaps and nuances between the key areas of concern identified. This part of the analysis suggested that as well as constituting a key concern in its own right, staff interpersonal skills were the most frequent driver of both positive and negative feedback, even in relation to other key areas of concern like treatment and staff communication skills. Other frequently cited drivers of positive and negative feedback included accessibility to care, patient-centredness, and staff-to-staff and staff-to-patient communication. Staff technical competence was a less prominent driver of feedback, cited only in relation to the evaluation of treatment itself, and accounting for a small minority of these comments. Our findings therefore support the notion that there is a discord between technical competence as it might be conceptualised and evaluated by practitioners and how it is conceptualised and evaluated by patients in their feedback on healthcare services.[16]

Strengths and weaknesses of the study

This study has examined the largest and most up-to-date collection of patient feedback on NHS services in England. Our use of quantitative computer-assisted linguistic techniques has produced large-scale, generalisable insights into this dataset. Yet at the same time, the more fine-grained, qualitative analysis was able to elucidate more specific concerns and areas of difference and overlap that have been overlooked by research employing solely quantitative approaches.

Although this dataset has proven to be a valuable resource for learning about individuals' concerns about the healthcare services they access, its lack of metadata regarding the demographic information of the contributors meant that it was not possible to attribute certain concerns to particular demographic groups. It is also worth bearing in mind that the

For peer review only

majority of the feedback contained in the data relates to the core areas of healthcare provision of GPs, hospitals and dentists. While this is unlikely to present issues respecting the general concerns examined, more specific concerns relating to these areas might be said to be over-represented compared to other areas of service provision, such as pharmacists, opticians and mental health trusts.

Strengths and weaknesses in relation to other studies and important differences in results

Where a great deal of existing research has explored patient feedback in terms of pre-determined themes or areas of concern, the data-driven approach adopted in the present study has allowed areas of concern to emerge from the comments themselves throughout the course of the analysis. As a consequence, issues relating to system and organisation, which remain largely unexplored in existing research, have emerged and been examined here not only as key concerns in their own right, but also as significant drivers of positive and negative evaluation with respect to various other aspects of care, including quality of treatment and staff communication skills.

As well as providing fresh insight into contemporary concerns for patients accessing healthcare services in England, the findings reported in this study also provide more substantive quantitative evidence to support and advance the findings reported in existing studies of patient feedback that are based on comparatively smaller and older datasets. Aresearch on patient feedback to-date.[17-18] However, our findings highlight the centrality of interpersonal skills not only as a key area of concern in the patient feedback, but also to the ways that treatment quality and staff communication are evaluated.

Meaning of the study: possible explanations and implications for clinicians and policymakers

While the majority of research into patient feedback has focused principally – in most cases exclusively – on what motivates negative feedback, the present study has elucidated the drivers of positive and negative feedback equally. Accordingly, while the reported drivers of negative feedback might flag up areas that require attention, the specific drivers of positive feedback outlined over the course of the analysis offer insight that can be used to stimulate and guide quality improvement efforts.[19-21]

Staff interpersonal skills lie central to improving care, as these have been shown to motivate both positive and negative feedback in relation to numerous areas of concern. The findings of this study suggest that developing the interpersonal skills of staff should be a priority in medical and non-medical staff training. The interpersonal skills of staff are evaluated

For peer review only

positively when staff are perceived as cheerful, compassionate and reassuring. Smiling, listening, having a good sense of humour and taking the time to learn the names of their patients are also viewed as important. Such skills are linked to the concept of emotional labour,[22] which involves the regulation of emotion to create a publicly visible facial and bodily display within the workplace. These kinds of attributes might seem more like individual character traits, and so incorporating these into training poses challenges, especially as members of the public are often unimpressed by 'scripted' interactions which are rightly seen as inauthentic.[23] However, other positively evaluated interpersonal aspects of care, such as involving patients in communication and decision making and ensuring that patients have sufficient time to interact with medical staff and are not made to feel as though they are "rushed", might constitute more tangible and attainable targets for skills development programs.

Another important finding from this study is the seeming significance of system issues relating to access to care. This often relates to technical difficulties experienced with online booking systems. Tightening government expenditure in healthcare provision and resultant constraints on practitioner time and availability mean that these issues are unlikely to abate. Such issues arguably lie within the remit of policy makers and governing bodies. However, practitioners and other staff can improve patient feedback in this area by making an effort to ensure that appointments run on time and informing and updating patients and their families in the case of cancellations or delays.

The findings reported here also provide insight into patient feedback more generally, and bear implications for how such feedback should be interpreted in the future. Our quantitative examination of the data revealed significant overlap between the drivers of positive and negative feedback across the areas of concern explored here. As an example, although Treatment fared best of these four areas of concerns in terms of positive evaluation, it was only by examining the comments relating to Treatment that we were able to show that 42 per cent of the positive comments relating to this concern actually praised interpersonal aspects of care, rather than the technical competence of staff, which accounted for only 12 per cent of these comments. It is therefore beneficial, where possible, to gage feedback at a granular level. This is where combining quantitative and qualitative approaches bears significant advantages for researchers, allowing us to deal with large datasets in a way that is sensitive to subtle nuances and overlaps and to point to specific areas for praise or improvement that only become apparent at the more granular level.

Unanswered questions and future research

Although the data contains feedback relating to a variety of areas of healthcare provision, such distinctions have not figured in the analysis undertaken in this study. Future studies on

For peer review only

this particular dataset should therefore take a modular, even comparative approach, to ascertain similarities or differences in the ways that care is evaluated in each of these areas. Furthermore, we did not have access to the demographic information of the comment posters. Future research should endeavour to collect and examine data that comprises this kind of demographic metadata in order to determine whether particular concerns are attributable to people living in certain locations or belonging to particular age, ethnic or sex related groups.

For peer review only

References

1 Vingerhoets E, Wensing M, Grol R. Feedback of patients' evaluations of general practice care: a randomised trial. *Qual Health Care* 2001;10:224–228.

2 Coulter A. Can patients assess the quality of health care? *BMJ* 2006; 333:1–2.

3 Evans RG, Edwards AG, Evans S, *et al.* Assessing the practising physician using patient surveys: A systematic review of instruments and feedback methods. *Family Practice* 2007;24:117–127.

4 Sitzia J. How valid and reliable are patient satisfaction data? An analysis of 195 studies. *International Journal for Quality in Health Care* 1999;11:319–328.

5 Davies E, Cleary PD. Hearing the patient's voice? Factors affecting the use of patient survey data in quality improvement. *BMJ Quality & Safety* 2005;14:428–432.

6 Veloski J, Boex JR, Grasberger MJ, *et al.* Systematic review of the literature on assessment, feedback and physicians' clinical performance: BEME Guide No. 7. *Medical Teacher* 2006;28:117–128.

7 Fitzpatrick R. Surveys of patient satisfaction: I-Important general considerations. *BMJ* 1991;302:887–889.

8 Gupta PC, Witty J, Wright N. An Approach to Consumer Feedback in an Out-patient Specialty Service. *International Journal of Health Care Quality Assurance* 1993;6:13 – 16.

9 Greco M, Brownlea A, McGovern J. Impact of patient feedback on the interpersonal skills of general practice registrars: results of a longitudinal study. *Medical Education* 2001;35:748–756.

10 Jung HP, Wensing M, Grol R. What makes a good general practitioner: do patients and doctors have different views? *British Journal of General Practice* 1997;47:805–809.

11 Lagu T, Hannon NS, Rothberg MB, *et al.* Patients' Evaluations of Health Care Providers in the Era of Social Networking: An Analysis of Physician-Rating Websites. *Journal of General Internal Medicine* 2010;25:942–946.

12 López A, Detz A, Ratanawongsa N, *et al.* What patients say about their doctors online: A qualitative content analysis. *Journal of General Internal Medicine* 2012;27:685–692.

13 Raleigh VS, Hussey D, Seccombe I, *et al.* Do associations between staff and inpatient feedback have the potential for improving patient experience? An analysis of surveys in NHS acute trusts in England. *Quality & Safety in Health Care* 2009;18:347–354.

14 Platonova EA, Kennedy KN, Shewchuk RM. Understanding patient satisfaction, trust, and loyalty to primary care physicians. *Medical Care Research and Review* 2008;65:696–712.

For peer review only

15 Hardie A. CQPweb — combining power, flexibility and usability in a corpus analysis tool. *International Journal of Corpus Linguistics* 2012;17:380–409.

16 Cheraghi-Sohi S, Bower P. Can the feedback of patient assessments, brief training, or their combination, improve the interpersonal skills of primary care physicians? A systematic review. *BMC Health Services Research* 2008;8:179.

17 Hall A, Dornan MC. Meta-analysis of satisfaction with medical care: description of research domain and analysis of overall satisfaction levels. *Social Science & Medicine* 1988;27:637–644.

18 Wensing M, Van der Weijden T, Grol R. Implementing guidelines and innovations in general practice: which interventions are effective? *British Journal of General Practice* 1998;48: 991–997.

19 Locker D, Dunt D. Theoretical and methodological issues in sociological studies of consumer satisfaction with medical care. *Social Science & Medicine* 1978;12:283-292.

20 Sitzia J, Wood N. Patient satisfaction: a review of issues and concepts. *Social Science & Medicine* 1997;45:1829–1843.

21 Cleary PD. The increasing importance of patient surveys. *BMJ* 1999; 319:720.

22 Hochschild A. *The Managed Heart: Commercialization of Human Feeling*. Berkeley: University of California Press, 1983.

23 Cameron D. *Good to Talk? Living and Working in a Communication Culture*. London: Sage, 2000.

For peer review only

Funding

Economic and Social Research Council; grant number: ES/K002155/1

Competing interests

All authors have completed the ICMJE uniform disclosure form and declare: all authors had financial support from the Economic and Social Research Council for the submitted work; no financial relationships with any organisations that might have an interest in the submitted work in the previous three years; no other relationships or activities that could appear to have influenced the submitted work."

Ethics approval

Research ethics approval for the study was obtained from Lancaster University, Lancaster, UK.

Contributorship statement

GB co-planned and co-conducted the research, and took charge of writing the paper. He is responsible for the content as guarantor. PB co-planned and co-conducted the research and contributed to the writing of the paper. Both authors had access to the data.

Exclusive licence

The Corresponding Author has the right to grant on behalf of all authors and does grant on behalf of all authors, a worldwide licence to the Publishers and its licensees in perpetuity, in all forms, formats and media (whether known now or created in the future), to i) publish, reproduce, distribute, display and store the Contribution, ii) translate the Contribution into other languages, create adaptations, reprints, include within collections and create summaries, extracts and/or, abstracts of the Contribution, iii) create any other derivative work(s) based on the Contribution, iv) to exploit all subsidiary rights in the Contribution, v) the inclusion of electronic links from the Contribution to third party material where-ever it may be located; and, vi) licence any third party to do any or all of the above.

Transparency statement

The lead author* affirms that this manuscript is an honest, accurate, and transparent account of the study being reported; that no important aspects of the study have been omitted; and that any discrepancies from the study as planned have been explained.

Data-sharing statement

No additional data available.

For peer review only

For peer review only

BMJ Open

What does patient feedback reveal about the NHS? A mixed methods study of comments posted to the NHS Choices Online service

Journal:	*BMJ Open*
Manuscript ID	bmjopen-2016-013821.R1
Article Type:	Research
Date Submitted by the Author:	21-Dec-2016
Complete List of Authors:	Brookes, Gavin; University of Nottingham, School of English<br>Baker, Paul; Lancaster University, Linguistics and English Language
**Primary Subject Heading**:	Communication
Secondary Subject Heading:	Health services research
Keywords:	Patient feedback, NHS Choices, Quantitative linguistics

SCHOLARONE™
Manuscripts

For peer review only

Title page

Title

What does patient feedback reveal about the NHS? A mixed methods study of comments posted to the NHS Choices Online service

Authors' names

Gavin Brookes, Paul Baker

Address for each author

Gavin Brookes
Research Fellow
Centre for Research in Applied Linguistics (CRAL), School of English, University of Nottingham, Nottingham, UK, NG7 2RD

Paul Baker
Professor of Linguistics and English Language
ESRC Centre for Corpus Approaches to Social Science (CASS), Department of Linguistics and English Language, Lancaster University, Lancaster, UK, LA1 4YL

Corresponding author

Gavin Brookes
Centre for Research in Applied Linguistics (CRAL)
School of English
University of Nottingham
Nottingham
UK
NG7 2RD
gavin.brookes@nottingham.ac.uk

For peer review only

Abstract

Objective
To examine the key themes of positive and negative feedback in patients' online feedback on NHS services in England and to understand the specific issues within these themes and how they drive positive and negative evaluation.

Design
Computer-assisted quantitative and qualitative study of 228,113 comments (28,971,142 words) of online feedback posted to the NHS Choices website. Comments containing the most frequent positive and negative evaluative words are qualitatively examined to determine key drivers of positive and negative feedback.

Participants
Contributors posting comments about the NHS between March 2013 and September 2015.

Results
Overall, NHS services were evaluated positively approximately three times more often than negatively. Four key areas of focus were: treatment, communication, interpersonal skills and system/organisation. Treatment exhibited the highest proportion of positive evaluative comments (87%), followed by communication (77%), interpersonal skills (44%) and finally system/organisation (41%). Qualitative analysis revealed that reference to staff interpersonal skills featured prominently, even in comments relating to treatment and system/organisational issues. Positive feedback was elicited in cases of staff being caring, compassionate and knowing patient's names while rudeness, apathy and not listening were frequent drivers of negative feedback.

Conclusions
Although technical competence constitutes an undoubtedly fundamental aspect of healthcare provision, staff members were much more likely to be evaluated both positively and negatively according to their interpersonal skills. Therefore, the findings reported in this study highlight the salience of such 'soft' skills to patients and emphasise the need for these to be focused upon and developed in staff training programs, as well as ensuring that decisions around NHS funding do not result in demotivated and rushed staff. The findings also reveal a significant overlap between the four key themes in the ways that care is evaluated by patients.

Strengths and limitations of this study
- This study examines the largest (228,113 comments and approximately 29 million words) and most recent (March 2013 to September 2015) collection of online patient comments on NHS services analysed to-date.
- Building on previous research, the feedback data examined relates to a wider range of areas of health care service provision, including: acute trusts, care organisations, care providers, clinical commissioning groups, clinics, dentists, GP practices, hospitals, mental health trusts, opticians and pharmacies. Although the comments relating to these various areas of provision are not compared in the analysis, this nonetheless makes for a more widely representative dataset.

For peer review only

- The use of quantitative computer-assisted linguistic techniques produces large-scale, generalisable insights into this vast dataset, while more fine-grained, qualitative analysis helps to elucidate nuances and areas of difference and overlap that have been overlooked by research employing solely quantitative approaches.
- Further data and research is required to assess possible demographic trends in the feedback given.

For peer review only

Introduction

Since the 1980s, patient feedback exercises have been undertaken by an increasing number of healthcare providers worldwide in order to monitor the quality of the services they provide and stimulate improvements where needed.[1] Although the reliability of patient feedback as an indicator of the technical quality of care remains a topic of debate,[2] patient feedback exercises have nonetheless become a staple way of measuring and regulating healthcare standards,[3,4] as well as ensuring public involvement in the design and improvement of healthcare provision.[5] Patient empowerment is, as Gann puts it, 'here to stay' (p. 150),[6] and policy makers the world over have come to recognise the potential of active patient involvement to drive service improvements, improve self-care, and ultimately improve the affordability and sustainability of the services they provide.[7] In England, since 2002 patient feedback has played an increasingly significant role in the way that care quality is assessed, with all National Health Service (NHS) trusts required to collect and report the results of feedback on their services to the regulatory body, the Healthcare Commission. The importance of the insights gained from patient feedback exercises is all the more pronounced in this context, where reductions in government expenditure in areas of social provision has required healthcare providers to constantly demonstrate both the quality and financial viability of the services they provide.

Healthcare providers can obtain feedback from their patients using a range of methods which can be implemented in different settings and at differing times following an episode of treatment. Ziebland and Coulter provide a list of such methods, which include (but are not limited to): face-to-face interviews, postal questionnaires, telephone interviewers (using automated and live interviewers), web-based online questionnaires, diaries, questions on hand-held portable devices, touch-screen kiosks, and bedside consoles.[8] Moreover, feedback can be collected on-site, at the point of service contact, or at patients' homes, some days, weeks or months later.[8] The analysis reported in this study focuses on feedback given in the form of online patient comments. In recent years, increasing attention has been paid, by researchers and healthcare providers, to the Internet as a site for patients to recount their express of healthcare services and to draw attention to what was good and bad about those experiences.[9-11] One such recent study was undertaken by Greaves and colleagues, who compared patients' ratings of care posted to the NHS Choices online service with the results of non-experiential measures of service performance, like morality rates.[12] The researchers reported that, overall, patients' ratings tended to correlate with the non-experiential measures. For example, hospitals that were poorly evaluated by patients were found to have higher mortality rates. This research therefore supports the value of online forms of patient feedback for assessing care quality and targeting areas for improvement.

Given the increasing significance of patient feedback to the ways that healthcare services are designed, delivered and regulated, there is a pressing need for research that accounts for the concerns expressed by patients in their feedback. However, rather than explore the content of patient feedback itself, the majority of existing research in this area is concerned chiefly with: reviewing the suitability of instruments and methods of collecting and analysing feedback,[13] considering the reliability of feedback data for assessing healthcare quality,[14] reflecting on the extent to which insights gained from such exercises have actually improved service provision,[15] and recommending how such insights might be translated into

For peer review only

positive clinical outcomes in the future.[16] The comparatively few studies that have examined the content of patient feedback (even fewer of which relate to healthcare in England), have reported recurring drivers of feedback to include the technical quality of the care, accessibility to care and the interpersonal and communication skills of practitioners (with the latter two often conflated).[17-19]

The present study identifies and examines the key drivers of positive and negative feedback on healthcare services given in patients' online comments posted to the NHS Choices website between March 2013 and September 2015. Our findings build on existing patient feedback research in several important ways. At 228,113 comments and approximately 29 million words, the feedback data we analyse is considerably larger than that examined in previous research on this topic, which has mainly accounted for hundreds of comments[20-22] and at the most in the tens of thousands.[23] Moreover, the data we analyse represents feedback relating to a wider range of healthcare services than considered in previous research, which has often focussed on specific areas of healthcare provision.[24] The lion's share of research on patient feedback was conducted using data collected in the 1990s and early 2000s, while our dataset contains comments made as recently as September 2015, making this dataset the most up-to-date of its kind. More broadly, given the ever changing landscape of healthcare provision in England and the U.K., the present study responds to the need for regular and up-to-date research that assesses patient attitudes towards the NHS and specifically identifies the key drivers of feedback about the particular services they access.

Methods

Data

We studied the written feedback posted to the NHS Choices online service (http://www.nhs.uk/pages/home.aspx) between March 2013 and September 2015 (data made available to the researchers). The comments were collected from the NHS Choices service's comprehensive RSS feed for posted comments, using a developer key provided to us for this purpose, and then converted from RSS/XML to suitable structured corpus/database format for analysis. The data comprises a total of 228,113 comments, amounting to 28,971,142 words. The comments relate to a variety of healthcare organisations, including acute trusts, care organisations, care providers, clinical commissioning groups, clinics, dentists, GP practices, hospitals, mental health trusts, opticians and pharmacies. However, the majority of the comments (27,005,715 = 93.21 per cent) relate to three primary care services: GP practices, hospitals and dentists.

Table 1. Breakdown of the NHS comments database

Section	No of comments	Word counts	Mean words per comment
Acute trusts	1,022	159,385	156
Care organisations	6	1,164	194
Care providers	4,493	422,133	94
CCGs	1	253	253

For peer review only - http://bmjopen.bmj.com/site/about/guidelines.xhtml

Clinics	2,887	400,813	139
Dentists	41,958	4,306,698	103
GP practices	111,318	14,093,437	127
Hospitals	55,145	8,605,580	157
Mental health trusts	565	111,557	197
Opticians	1,734	179,493	104
Pharmacies	8,984	690,629	77
**All sections**	**228,113**	**28,971,142**	**146**

For peer review only

Analysis

We examined the comments using computer-assisted methods of linguistic analysis afforded by CQPWeb,[25] an online tool that offers a range of techniques for quantitatively and qualitatively analysing large collections of digitised language data. We began by identifying the ten most frequently occurring linguistic markers of positive and negative evaluation across the comments. These words were manually identified from a list of all the words occurring in the data provided by the 'frequency' function of CQPWeb. Evaluation is a complex linguistic phenomenon, and can be made according to a variety of parameters, including the extent to which things are important, expected, comprehensible, possible and reliable. To ensure that our analysis captured the broadest range of themes concerning the positive and negative evaluation in the comments, we focused on the most generic evaluative items, i.e. words that were broadly used to describe something as either being good or bad.

Using CQPWeb we then generated a list of those words that tend to occur frequently alongside the positive and negative evaluative words in the comments, that is, their most frequent 'collocates'. Collocation refers to 'the characteristic co-occurrence patterns of words'.[26] By analysing the collocates of the evaluative words, we were able to get a sense of what tended to be the target of the evaluation in the feedback – i.e. of what was evaluated as 'good' and 'bad' in the comments. These words therefore reflect key themes of positive and negative feedback in the data.

Building on this, the next, more qualitative step in our procedure involved closely reading a randomly-selected sample of comments in which each theme was evaluated positively and negatively to determine the more specific reasons or 'drivers' of the evaluation. Each sample consisted of 100 comments and contained comments relating to all organisations represented in the data. To ensure that 100 comments provided a sufficiently representative sample for this stage in our analysis, we adopted a saturation point procedure, well established in such quantitative linguistic research,[27] of randomly selecting 30 comments, analysing the emergent patterns, proceeding to examine another 30 randomly selected comments, and continuing the process until saturation point was reached and new patterns had ceased to emerge. New patterns, or drivers, were no longer emergent by the time we analysed the 100th comment (positive and negative) for each theme, and so this sample size was deemed sufficiently large to account for the common drivers of positive and negative feedback, yet small enough to facilitate fine-grain qualitative examination.

Results

Quantitative findings

Table 2 displays the ten most frequent positive and negative evaluative words used in the comments. The comparatively higher frequencies of the positive words (total: 223,439) compared to the negative words (total: 73,363) provides a quantitative indication that the patients are more likely to evaluative the services they access positively than negatively. The positive evaluation words occur, on average, across almost three times as many comments as the negative words.

Table 2. Ten most frequent positive and negative evaluative words in the comments

Positive			Negative		
**Word**	**Frequency**	**# of comments**	**Word**	**Frequency**	**# of comments**
good	59,237	46,192	bad	16,945	14,798
excellent	49,090	38,128	poor	15,274	12,548
great	34,298	27,772	worst	7,627	6,627
best	25,556	21,641	worse	7,289	6,447
fantastic	15,186	12,915	terrible	6,799	5,920
brilliant	11,546	10,136	awful	6,106	5,291
wonderful	10,371	8,846	appalling	4,410	4,007
amazing	9,749	8,081	disgusting	3,246	2,913
outstanding	5,019	4,277	ridiculous	3,206	3,023
exceptional	3,387	3,060	useless	2,461	2,217
**Total**	**223,439**	**18,105 (mean)**	**Total**	**73,363**	**6,379 (mean)**

CQPWeb was then used to generate lists of those words occurring frequently within the three words preceding and following the positive and negative evaluation words in table 2 (i.e. their collocates). The collocational span of 3 is the default value in CQPWeb and is fairly standard in collocation analyses, which tend to operate with spans ranging from 3 to 5 words.[28] As we mentioned earlier, evaluation is a complex linguistic phenomenon, and the positive words below could, in some circumstances, be used to evaluate something negatively ('not good'), and vice versa (e.g. 'isn't bad'). Such cases comprised a tiny proportion (under 1%) of cases and were removed from the remainder of the analysis below.

The 100 most frequent words occurring alongside the positive and negative words were thematically coded to reflect the most frequently evaluated areas of concern for patients giving feedback. Four areas emerged as frequent across the comments (corresponding

For peer review only

words in brackets): (i) treatment (*care, treatment*, *dental*), (ii) communication (*communication, attention, listener(s), advice*), (iii) interpersonal skills (*atmosphere*, *attitude(s), manner(s)*) and (iv) system/organisation (*system*, *appointment*, *management* and *waiting time(s)*). As the forthcoming qualitative analysis shall demonstrate, feedback concerning communication and interpersonal skills related to a mixture of medical and non-medical staff groups, with the latter including staff members like receptionists and managers. Note that we combined *waiting* and *time(s)* together into one linguistic item, as references to *time(s)* by itself often appeared in statements like 'I had a really bad time', which were too vague to be categorised. Based on the corresponding words (in brackets), we then examined how often each concern featured alongside the positive vs. negative evaluative words (figure 1).

Figure 1. Collocation of most frequent feedback themes with positive and negative evaluation words
<INSERT FIGURE 1 HERE>

Of the four key themes displayed above, treatment exhibited the highest proportion of positive feedback, occurring alongside the positive evaluation words 87 per cent of the time. Communication was also evaluated positively overall (77%), while interpersonal skills were only evaluated positively 44% of the time and system/organisational issues fared worst of all with only a 41% positive evaluation.

Qualitative findings

To understand why the four key themes identified in our quantitative analysis were evaluated positively and negatively across the patients' comments, we examined a sample of comments (n= 100) in which each occurred alongside the positive and then negative evaluation words. The ensuing section will report the reasons each theme was positively and negatively evaluated in our sample, dealing with each theme in turn, starting with the theme that fared best in the patients' comments (treatment), and concluding with the theme that fared worst (system/organisation).

Treatment

Table 3. Reasons treatment was positively evaluated

Category	Percentage of sample	Example comment
Good interpersonal skills	47%	Excellent Dental Care and service, Highly recommend I have been a patient at [name] for approx 8 years, and thought I would drop a line to say how satisfied I am with the full service the practice provides. The staff are friendly, polite and professional and provide sufficient information at all times.

For peer review only

Good communication skills	16%	Always great treatment, reception staff are also very good at explaining all options. Cannot recommend the team highly enough.
Technically competent	10%	Excellent customer care Excellent service from a friendly knowledgeable team.
Patient-centred	9%	The dental care is excellent treating one with respect and discussing with you treatment options. I would particularly recommend the senior nurse who provides a very individualised care.
Efficient	8%	Good Surgery I am a member of the health service and I have to say that everyone at the surgery is very helpful and friendly and the treatment I have received has been very efficient and effective in dealing with my concerns.
Hard-working	5%	I don't think the people who have made negative comments understand how difficult it can be for a GP practice to try and meet the demands of today's patients, what you have here is a committed team of GPs, nurses and admin staff who are working hard daily to give patients their best service and care.
Clean facilities	3%	I received wonderful care during my 6 weeks there and it was very clean too.
Good food	2%	After my surgery was done the nurses took great care of me and the food was 1st rate.

Table 4. Reasons treatment was negatively evaluated

Category	Percentage of sample	Example comment
Poor interpersonal skills	35%	Nursing staff are insensitive, they have poor insight in how to care for the mentally ill. They are bullies and self-centred. Patients are picked on and have the micky taken out of them by nursing staff. An absolute shambles and one of the worse care providers.
Lack of appointment availability	25%	Not only can you never get an appointment, but the extremely rude and unhelpful receptionist tops this GP surgery to be surely one of the worst for

For peer review only

		care of patients.
Technically incompetent	18%	Ward 23 Appalling care. Our relative was admitted for simple antibiotics for an infection in her leg. 2 days later she is bed bound after having her other medical complications completely ignored.
Poor communication skills	8%	Simple nursing care non-existent, communication to family again non-existent.
Lack of aftercare	6%	Aftercare seems to be something they have no idea exists... instead of being a standard procedure it is totally ignored. That tells me they do not care. Absolutely Awful - If you can go to a different GP then do!
Unclean facilities	4%	The place is dirty and the standard of care is so bad I honestly can't believe the hospital has not been shut down.
Difficulty accessing test results	2%	Poor customer care. The GPs themselves are good. However, I had blood tests done recently and have had a nightmare trying to find out the results.
Lack of seating and space in waiting areas	2%	People that were visibly ill were holding themselves up against the walls and sitting on the floor. It was absolutely shocking. Probably the worst care I have ever seen within the NHS. Again, no further seating was provided

Communication

Table 5. Reasons communication was positively evaluated

Category	Percentage of sample	Example comment
Listens to patients	44%	He is an extremely skilled dentist who pays great attention to detail and to listening to his patients' concerns.
Good interpersonal skills	29%	The most best gp I have ever met , so nice to talk , very patience dr, more professional, very good listener very helpful, very kind!
Good communication between staff members	8%	Excellent Team The [NAME] Practice offers an excellent team approach to

For peer review only

		healthcare, as far as I can see all services are fully integrated with excellent communication between all disciplines.
Not rushed	7%	I have found my doctor to be a very good listener and though doctors are very busy, I have never felt my consultations rushed.
Sensitive	5%	Communication was excellent, given in a sensitive caring manner and straightforward clarity.
Frequent	3%	Communication wad excellent and I was always kept up to date by my surgeon.
Involves patient's family	3%	The communication was excellent with my husband being able to ask questions about the timing of the surgery, what to expect & how he could help in my recovery!
Perceived as honest	1%	This to me is the best NHS GP surgery in the area! Caring and second to none treatment honest good advice.

Table 6. Reasons communication was negatively evaluated

Category	Percentage of sample	Example comment
Doesn't listen to patients	32%	Patronising and a bad listener I have decided to register with another GP after being a patient at this clinic for 4 year. I feel that one doctor doesn't really take the time to listen, even if it is a problem that may be sensitive or embarrassing.
Poor interpersonal skills	29%	The two doctors have bad attitude and very bad advice and clearly don't care about your welfare. Totally avoid.
Poor inter-staff communication	14%	There is poor communication between admin here and medical staff. Results go into a black hole and don't seem to reach or be reviewed or followed up by doctors.
Failure to make contact to arrange appointments	9%	We waited 6 weeks to receive an appointment for our son despite continued phone calls to the consultants secretary and a business manager and various admin staff. NTW obviously feel at this level of communication is adequate. When I did ring on numerous

For peer review only

		occasions I was assured a response but not rang back.
Unreliable telephone services	7%	My complaint is in relation to poor communication from outside agencies and from myself trying to access support via telephone. There is an online service but that advises you not to leave anything important as it is not always checked.
Failure to involve patients' relatives	4%	However after another phone call to the A & E sister she explained very clearly that my mother had in actual fact gone from them to the [NAME] ward at around 04.30 and they did not want to contact me at that early time but expected the ward staff to do this after admission to their ward. The Sister agreed that it was poor communication, especially as my mother is very elderly and frail and potentially having fractured her hip she may have died.
Rushed	4%	Rushed consultations and poor communication.
Poor English language skills	1%	Front line staff are poor in communication and poorly trained except for the two senior staff. some of the staff at the reception are unable to express (in English) causing miscommunication and stress to patients.

Interpersonal skills

Table 7. Reasons interpersonal skills were positively evaluated

Category	Percentage of sample	Example comment
Friendly and approachable	41%	Some emergency Dentists treat people with indifference but not this one, lovely warm people, great atmosphere, many thanks.
Good communication with patients	27%	The doctor I saw had an excellent manner. Answered all my questions. Wonderful service.
Listens to patients' concerns	11%	On the positive, the doctor has a great manner with their patients and takes time to listen to your story and

For peer review only

		symptoms.
Empathetic	9%	They were brilliant and really cared; they had a fantastic bedside manner and is genuinely empathetic.
Patient-centred	7%	The doctors "bedside manner" is great. They take time to listen to concerns and talk through clearly and carefully the options to deal with my issues and problems.
Smiles	3%	The nursing staff were very friendly and helpful, and with a very good attitude, always smiling which I imagine would go a long way to helping relieve any nerves some people may have.
Good sense of humour	2%	Can't fault the practice, courteous service good communication, all done with a relaxed manner and good humour.

Table 8. Reasons interpersonal skills were negatively evaluated

Category	Percentage of sample	Example comment
Rude/impolite	54%	Receptionist staff are disrespectful and have a complete bad attitude towards me and others over the phone, some may be kind but other than that they have a disgusting attitude, as for doctors, they are ok since they listen to me.
No one answers the phone	16%	Extreme bad manners when you have to call the surgery 8 times (and counting) to ask to speak to a doctor (during the times they recommend)
Dismissive	11%	Bad manners Reception have a terrible attitude, very rude and dismissive.
Lazy	11%	Worse still when I try and book a later date the only GP that is available is awful. Their attitude is diabolical and the snorting all the time is so off putting. I find them so rude and their lethargic attitude annoys me.
Doesn't listen to patients	5%	Never have I witnessed such bad manners from a healthcare professional. Did not listen or care about anything I had to say.
Not smiling/look unhappy	3%	One of the drs for children is especially bad attitude , they always showing unhappy face to my children who is

For peer review only

		afraid , and they were rude to my partner one time when he was not speaking good English.

System and organisation

Table 9. Reasons system and organisational issues were positively evaluated

Category	Percentage of sample	Example comment
Good appointment availability	30%	Excellent appointment availability I understand this surgery is a training practice which is supervised by permanent senior doctors.
Good appointment booking system	25%	The online appointment system is excellent and takes pressure off phone lines and means that it is easier to find an appointment that suits, with a preferred GP If need be.
Short waiting times	23%	Very pleased [name] ward - Great hygiene, friendly members of staff, good waiting times, reassuring and good with patients.
Good telephone services	11%	The doctor call back system is an excellent use of resources and is a good triage system ensuring that a patient is seen by the appropriate person.
Good availability of emergency appointments	8%	I have always been able to get an appointment in an emergency in good time.
Good prescription system	3%	I use the new on line system which is excellent for requesting repeat prescriptions

Table 10. Reasons system and organisational issues were negatively evaluated

Category	Percentage of sample	Example comment
Poor appointment availability	27%	Poor appointment availability I tried to make an appointment for four days ahead but was told all appointments on that day were embargoed i.e. not available, and so I was advised to phone back next day.
Long waiting times	22%	Waiting time awful I had a scheduled appointment and waited for more than 1.5 hours and still I was not seen by the doctor, nor was I given any explanation or

For peer review only

		information as to when I would be seen.
Poor appointment booking system	19%	The appointment system very bad and the phone is constantly engaged and when you do ring for an appointment there is a silly system there that only takes 3 days advance booking
Long telephone waiting times	17%	The telephone system is awful, I am told to press zero to speak to someone then told to ring back later by an automated voice because they are busy. This costs me money to ring and is very frustrating.
Poor appointment time management	10%	Terrible long waiting time for appointments Every time I go in to see the GP, I have to wait at least 40mins after my appointment time to see a doctor, it's really annoying that time is not managed effectively here.
Limited opening hours	3%	Wake up Mere Lane modernise your service to the standard of your Building & Doctors Great doctors, great facilities, awful appointment service & opening hours!
Lack of availability of emergency appointments	2%	Staff are very rude and not interested in your wellbeing, telephone appointments are at a far reach as its always fully booked , no surprise there as there opening hours are awful. Even for an emergency appointment.

Discussion

Statement of principal findings

The online comments analysed in this study paint a generally positive picture of healthcare services provided by the NHS in England. Our quantitative analysis of the patient comments revealed the most commonly used linguistic markers of positive evaluation occurred approximately three times as often as markers of negative evaluation and, on average, across approximately three times as many comments. Patients' experiences and impressions of their treatment, communication, staff members' interpersonal skills and system/organisational issues were identified as key to the ways that healthcare services were both positively and negatively evaluated. Of these key themes, treatment exhibited the highest proportion of positive feedback, occurring alongside the positive evaluation words 87 per cent of the time. Communication was also evaluated positively overall (77%), while interpersonal skills were only evaluated positively 44% of the time and system/organisational Issues fared worst of all with only a 41% positive evaluation.

For peer review only

Qualitative examination of the data was able to reveal the more precise nature of the evaluation made in the comments, as well as uncover overlaps and nuances between the key themes of positive and negative feedback. This part of the analysis suggested that as well as constituting a key theme in its own right, staff members' interpersonal skills also emerged as a frequent driver of both positive and negative feedback in relation to how treatment and staff communication skills were evaluated. Other frequently cited drivers of positive and negative feedback included accessibility to care, patient-centredness, and staff-to-staff and staff-to-patient communication. Staff technical competence was a less prominent driver of feedback, cited only in relation to the evaluation of treatment itself, and accounting for a relative minority of these comments. Our findings therefore support the notion that there is a discord between the significance that practitioners and patients place on technical competence when judging the overall quality of care.[29]

Strengths and weaknesses of the study

This study has examined the largest and most up-to-date collection of patient feedback on NHS services in England in any format. Our use of quantitative computer-assisted linguistic techniques has produced large-scale, generalisable insights into this dataset. Yet at the same time, the more fine-grained, qualitative analysis was able to elucidate areas of difference and overlap that have been overlooked by research employing solely quantitative approaches in the past.

Although this dataset has proven to be a valuable resource for learning about individuals' perspectives on the healthcare services they access, its lack of metadata regarding the demographic information of individual contributors meant that it was not possible to attribute particular types of comment or concern to any demographic group. It is also worth bearing in mind that the majority of the comments we analysed (93.21 per cent) relate to the primary care services of GPs, hospitals and dentists. While this is unlikely to present issues respecting the general trends examined, more specific comments relating to these areas might be said to be over-represented compared to other areas of service provision, such as care providers and mental health trusts. Moreover, the data analysed in this study represents feedback given in one specific form (online comments), posted to one particular website (NHS Choices), about organisations based in one country (England). This raises issues surrounding representativeness, for those who choose to share their experiences online are not necessarily representative of the general population. It is now well-documented that, compared to non-Internet users, Internet users tend to be younger, more educated and from higher income brackets.[30] Although this digital divide is estimated to have narrowed over time,[11] the perspectives of people from these so-called 'hard-to-reach' or 'seldom-heard' groups are still likely to be under-represented in our data.[8,31]

Strengths and weaknesses in relation to other studies and important differences in results

Where a great deal of existing research has explored patient feedback in terms of pre-determined themes, the data-driven approach adopted in the present study has allowed drivers of feedback to emerge from the comments themselves throughout the course of the

For peer review only

analysis. As a consequence, system and organisation issues, which have remained largely unexplored in existing research, have emerged here as significant drivers of positive and negative evaluation with respect to various other aspects of care, including quality of treatment and staff communication skills.

As well as providing fresh insight into the perspectives of patients accessing contemporary healthcare services in England, the findings reported in this study also provide more substantive quantitative evidence to support the findings reported in existing studies of patient feedback that are based on comparatively smaller and older datasets.[32,33] However, our findings highlight the centrality of interpersonal skills not only as a key area of concern in its own right, but also as significant to the ways that treatment quality and staff communication are evaluated.

Meaning of the study: possible explanations and implications for clinicians and policymakers

While the majority of research into patient feedback has focused principally – in most cases exclusively – on what motivates negative feedback, the present study has elucidated the drivers of positive and negative feedback equally. Accordingly, while the reported drivers of negative feedback might flag up areas that require attention, the specific drivers of positive feedback outlined over the course of the analysis offer insight that can be used to stimulate and guide quality improvement efforts.[34-36]

The quantitative section of our analysis suggested that system and organisational issues to be a prominent theme in negative feedback. This often relates to issues surrounding accessibility of care, such as (emergency) appointment availability, waiting times, technical difficulties experienced with online booking systems, telephone waiting times and practice opening times. Tightening government expenditure in healthcare provision and resultant constraints on practitioner time and availability mean that these issues are unlikely to abate. Such issues arguably lie within the remit of policy makers and governing bodies. However, practitioners and other staff can improve patient feedback in this area by making an effort to ensure that appointments run on time and informing and updating patients and their families in the case of cancellations or delays.

The qualitative section of our analysis suggests that staff interpersonal skills lie central to improving care, as these were shown to motivate negative (and positive) feedback in relation to a variety of areas of concern. On the surface, the findings of this study suggest that developing the interpersonal skills of staff should be a priority in staff training. The interpersonal skills of both medical and non-medical staff were evaluated positively for qualities like being friendly and approachable, empathetic, for smiling, and not being afraid to laugh and joke with patients. Allied to this, practitioners were frequently positively evaluated for providing care that was patient-centred and involved discussing treatment options with patients, as well as explaining treatment plans and listening to their concerns. Conversely, staff were negatively evaluated when they were perceived as being rude, dismissive, lazy, not listening to patients' concerns, as well as for not smiling and appearing unhappy.

For peer review only

Most professional medical training (e.g. in medicine and nursing) includes the development of communication skills as a key element at the undergraduate level and onwards. This kind of interpersonal training is often focused on developing skills like information-gathering and shared decision making. Our findings suggest that, as far as patients are concerned, the interpersonal aspect of interaction is given a high premium. Such skills might be developed more effectively through greater opportunity for hands-on human engagement, rather than instruction alone, at the early stages of training. In terms of developing the interpersonal skills of other, non-medical staff groups, it is likely that many staff working in administrative capacities, such as receptionists, will not have received formal training interpersonal skills (although healthcare providers are increasingly running courses in 'customer service' to address this). Likewise, there is an intermediate group, which includes staff working in healthcare assistance, who may have received limited or no formal training, but who nonetheless engage with patients at very significant levels and may benefit from some form of interpersonal skills training.

However, many of these specific interpersonal or 'soft' skills can be linked to the concept of emotional labour,[37] which involves the regulation of emotion to create a publicly visible facial and bodily display within the workplace. These kinds of attributes might seem more like individual character traits, and so incorporating these into training poses challenges, especially as members of the public are often unimpressed by 'scripted' interactions which are rightly seen as inauthentic.[38] Soft skills training, while clearly helpful in some areas, may sometimes be a 'sticking plaster' solution to cover for wider structural problems involving over-stretched systems. Other positively evaluated interpersonal aspects of care, such as involving patients in communication and decision making and ensuring that patients have sufficient time to interact with medical staff and are not made to feel as though they are "rushed", might constitute more tangible and attainable targets for skills development programs.

In an effort to stimulate such improvements, the findings from the project from which this research derives, including the results reported in this article, have been presented to the Insight and Feedback Team at NHS England as well as the Care Quality Commission in the UK. However, in reality, translating such findings into practice is seldom straightforward. After all, although most major public sector healthcare providers collect feedback on their services at least annually, this information is not always used to improve service quality.[3] Suggested reasons for this include a lack of attention to patients' experience at senior levels,[39] as well as feedback data not being specific to particular wards or teams.[40]

As well as gesturing towards areas for improvement in healthcare provision, the findings reported here also provide insights into patient feedback more generally; insights which likely bear implications for how such feedback should be interpreted in the future. Our quantitative examination of the data revealed significant overlap between the drivers of positive and negative feedback. As an example, although treatment fared best of these four themes of feedback in terms of positive evaluation, it was only by examining the comments relating to treatment that we were able to show that 47 per cent of the positive comments relating to this theme actually praised interpersonal aspects of care, rather than the technical competence of staff, which accounted for only 10 per cent of these comments. It is

For peer review only

therefore beneficial, where possible, to gage feedback at a granular level. This is where combining quantitative and qualitative approaches can bear significant advantages for researchers, allowing us to deal with large datasets in a way that is sensitive to subtle nuances and overlaps and to point to specific areas for praise or improvement that only become apparent at the more granular level.

Unanswered questions and future research

Although the data contains feedback relating to a variety of healthcare organisations, such distinctions have not figured in the analysis undertaken in this study. Future studies on this particular dataset should therefore take a modular, even comparative approach, to ascertain similarities or differences in the ways that care is evaluated in each of these areas. Furthermore, we did not have access to the demographic information of the comment posters. Future research should endeavour to collect and examine data that comprises this kind of demographic metadata in order to determine whether particular concerns are attributable to people living in certain locations or belonging to particular age, ethnic or sex related groups. Future research should also assess feedback given in other, particularly non-digital, mediums in order to help to account for the perspectives of patients from 'hard-to-reach', 'seldom-heard' groups who might be less likely to give feedback online. Ziebland and Coulter recommend that non-traditional methods of data collection, such as pictures, stories and drama might be used to incorporate the views of such groups in future feedback collection exercises.[8]

References

1 Vingerhoets E, Wensing M, Grol R. Feedback of patients' evaluations of general practice care: a randomised trial. *Qual Health Care* 2001;10:224–228.

2 Coulter A. Can patients assess the quality of health care? *BMJ* 2006;333:1–2.

3 Graham C, Woods P. Patient experience surveys. In: Ziebland S, Coulter A, Calbrese J D, Locock L, eds. Understanding and Using Health Experiences: Improving patient care. Oxford: Oxford University Press 2013:81–93.

4 Swick H M. Toward a normative definition of medical professionalism. *Academic Medicine* 2000; 75:612–616.

5 Coulter A. Understanding the experience of illness and treatment. In: Ziebland S, Coulter A, Calbrese J D, Locock L, eds. Understanding and Using Health Experiences: Improving patient care. Oxford: Oxford University Press 2013:6–15.

6 Gann B. Understanding and using health experiences: the policy landscape. In: Ziebland S, Coulter A, Calbrese J D, Locock L, eds. Understanding and Using Health Experiences: Improving patient care. Oxford: Oxford University Press 2013:150–161.

7 Coulter A. Engaging patients in healthcare. Maidenhead: Open University Press 2011.

8 Ziebland S, Coulter A. Introduction. In: Ziebland S, Coulter A, Calbrese J D, Locock L, eds. Understanding and Using Health Experiences: Improving patient care. Oxford: Oxford University Press 2013:1–5.

For peer review only

9 Robinson K M. Unsolicited narratives from the internet: a rich source of qualitative data. *Qualitative Health Research* 2001;11:706–714.

10 O'Brien M R, Clark D. Unsolicited written narratives as a methodological genre in terminal illness. *Qualitative Health Research* 2012;22:274–284.

11 Mazanderani F, Powell J. Using the Internet as a source of information about patients' experiences. In: Ziebland S, Coulter A, Calbrese J D, Locock L, eds. Understanding and Using Health Experiences: Improving patient care. Oxford: Oxford University Press 2013:94–103.

12 Greaves F, Pape U J, King D, Darzi A, Majeed A, Wachter R M, Millett C. Associations Between Web-Based Patient Ratings and Objective Measures of Hospital Quality. *Archives of Internal Medicine*;172:435–436.

13 Evans RG, Edwards AG, Evans S, *et al.* Assessing the practising physician using patient surveys: A systematic review of instruments and feedback methods. *Family Practice* 2007;24:117–127.

14 Sitzia J. How valid and reliable are patient satisfaction data? An analysis of 195 studies. *International Journal for Quality in Health Care* 1999;11:319–328.

15 Davies E, Cleary PD. Hearing the patient's voice? Factors affecting the use of patient survey data in quality improvement. *BMJ Quality & Safety* 2005;14:428–432.

16 Veloski J, Boex JR, Grasberger MJ, *et al.* Systematic review of the literature on assessment, feedback and physicians' clinical performance: BEME Guide No. 7. *Medical Teacher* 2006;28:117–128.

17 Fitzpatrick R. Surveys of patient satisfaction: I-Important general considerations. *BMJ* 1991;302:887–889.

18 Gupta PC, Witty J, Wright N. An Approach to Consumer Feedback in an Out-patient Specialty Service. *International Journal of Health Care Quality Assurance* 1993;6:13 – 16.

19 Greco M, Brownlea A, McGovern J. Impact of patient feedback on the interpersonal skills of general practice registrars: results of a longitudinal study. *Medical Education* 2001;35:748–756.

20 Jung HP, Wensing M, Grol R. What makes a good general practitioner: do patients and doctors have different views? *British Journal of General Practice* 1997;47:805–809.

21 Lagu T, Hannon NS, Rothberg MB, *et al.* Patients' Evaluations of Health Care Providers in the Era of Social Networking: An Analysis of Physician-Rating Websites. *Journal of General Internal Medicine* 2010;25:942–946.

22 López A, Detz A, Ratanawongsa N, *et al.* What patients say about their doctors online: A qualitative content analysis. *Journal of General Internal Medicine* 2012;27:685–692.

23 Raleigh VS, Hussey D, Seccombe I, *et al.* Do associations between staff and inpatient feedback have the potential for improving patient experience? An analysis of surveys in NHS acute trusts in England. *Quality & Safety in Health Care* 2009;18:347–354.

24 Platonova EA, Kennedy KN, Shewchuk RM. Understanding patient satisfaction, trust, and loyalty to primary care physicians. *Medical Care Research and Review* 2008;65:696–712.

25 Hardie A. CQPweb — combining power, flexibility and usability in a corpus analysis tool. *International Journal of Corpus Linguistics* 2012;17:380–409.

26 McEnery T, Xiao R, Tono Y. Corpus-based language studies: An advanced resource book. London and New York: Routledge 2006.

27 Sinclair J. Reading concordances. London: Longman 2003.

For peer review only

28 Baker, P. Using Corpora in Discourse Analysis. London: Continuum 2006.

29 Cheraghi-Sohi S, Bower P. Can the feedback of patient assessments, brief training, or their combination, improve the interpersonal skills of primary care physicians? A systematic review. *BMC Health Services Research* 2008;8:179.

30 Norris P. Digital Divide: Civic Engagement, Information Poverty, and the Internet Worldwide. Cambridge: Cambridge University Press 2001.

31 Ryan S. Engagement and Inclusivity in researching patients' experiences. In: Ziebland S, Coulter A, Calbrese J D, Locock L, eds. Understanding and Using Health Experiences: Improving patient care. Oxford: Oxford University Press 2013:127–137.

32 Hall A, Dornan MC. Meta-analysis of satisfaction with medical care: description of research domain and analysis of overall satisfaction levels. *Social Science & Medicine* 1988;27:637–644.

33 Wensing M, Van der Weijden T, Grol R. Implementing guidelines and innovations in general practice: which interventions are effective? *British Journal of General Practice* 1998;48: 991–997.

34 Locker D, Dunt D. Theoretical and methodological issues in sociological studies of consumer satisfaction with medical care. *Social Science & Medicine* 1978;12:283-292.

35 Sitzia J, Wood N. Patient satisfaction: a review of issues and concepts. *Social Science & Medicine* 1997;45:1829–1843.

36 Cleary PD. The increasing importance of patient surveys. *BMJ* 1999; 319:720.

37 Hochschild A. *The Managed Heart: Commercialization of Human Feeling*. Berkeley: University of California Press, 1983.

38 Cameron D. *Good to Talk? Living and Working in a Communication Culture*. London: Sage, 2000.

39 Robert G, Cornwell J, Brearley S, Foot C, Goodrich J, Joule N, Levenson R, Maben J, Murrells T, Tsianakas V, Waite D. 'What matters to patients'? Developing the evidence base for measuring and improving patient experience. London: NHS Institute for Innovation and Improvement 2011.

40 Reeves R, Seccombe I. Do patient surveys work? The influence of a national survey programme on local quality-improvement initiatives. *Quality and Safety in Health Care* 2008;17:437–441.

Funding

Economic and Social Research Council; grant number: ES/K002155/1

Competing interests

All authors have completed the ICMJE uniform disclosure form and declare: all authors had financial support from the Economic and Social Research Council for the submitted work; no financial relationships with any organisations that might have an interest in the submitted work in the previous three years; no other relationships or activities that could appear to have influenced the submitted work."

Ethics approval

Research ethics approval for the study was obtained from Lancaster University, Lancaster, UK.

For peer review only

Contributorship statement

GB co-planned and co-conducted the research, and took charge of writing the paper. He is responsible for the content as guarantor. PB co-planned and co-conducted the research and contributed to the writing of the paper. Both authors had access to the data.

Exclusive licence

The Corresponding Author has the right to grant on behalf of all authors and does grant on behalf of all authors, a worldwide licence to the Publishers and its licensees in perpetuity, in all forms, formats and media (whether known now or created in the future), to i) publish, reproduce, distribute, display and store the Contribution, ii) translate the Contribution into other languages, create adaptations, reprints, include within collections and create summaries, extracts and/or, abstracts of the Contribution, iii) create any other derivative work(s) based on the Contribution, iv) to exploit all subsidiary rights in the Contribution, v) the inclusion of electronic links from the Contribution to third party material where-ever it may be located; and, vi) licence any third party to do any or all of the above.

Transparency statement

The lead author* affirms that this manuscript is an honest, accurate, and transparent account of the study being reported; that no important aspects of the study have been omitted; and that any discrepancies from the study as planned have been explained.

Data-sharing statement

No additional data available.

Acknowledgements

We wish to thank Dick Churchill for his advice concerning the pedagogical implications of our findings.

For peer review only

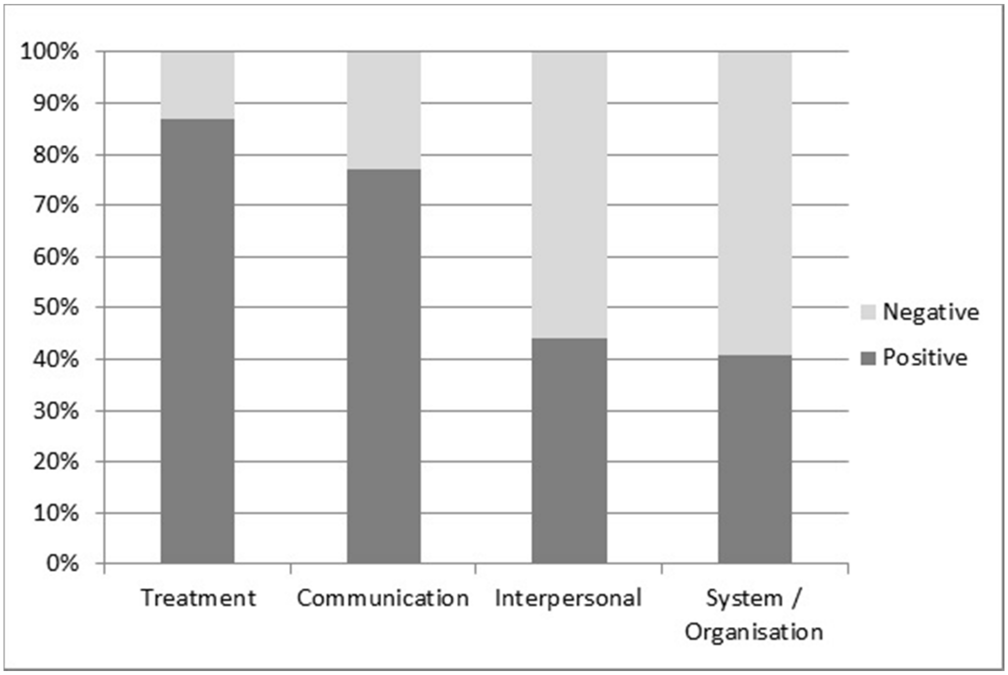

Figure 1. Collocation of most frequent feedback themes with positive and negative evaluation words

157x105mm (96 x 96 DPI)

view only
